# Boosting Boron Neutron Capture Therapy through Ferroptosis Activation with a Biomimetic Nanoenhancer

**DOI:** 10.34133/bmr.0343

**Published:** 2026-04-03

**Authors:** Jiayi Wang, Linwen Lv, Hao Li, Xin Pan, Junhui Zhang, Jiaxuan Li, Ruyu Yan, Jiaxin Wan, Haojun Liang, Bingbing Peng, Mingxin Yang, Yanan Chang, Juan Li, Hui Yuan, Minghua Shen, Gengmei Xing, Kui Chen

**Affiliations:** ^1^ Medical College of Yanbian University, Yanji133002, Jilin, China.; ^2^ CAS Key Lab for Biomedical Effects of Nanomaterials and Nanosafety, Institute of High Energy Physics, Chinese Academy of Sciences, Beijing 100049, China.

## Abstract

Boron neutron capture therapy (BNCT) is an emerging tumor radiotherapeutic modality, but its efficacy is limited by incomplete understanding of tumor cell death mechanisms. Here, we demonstrate that BNCT fails to sufficiently induce ferroptosis—a form of programmed cell death with important implications for both tumor elimination and immune activation—which constrains its therapeutic potential. Using proteomic profiling across multiple tumor cell lines, we found that although BN-triggered mitochondrial dysfunction activated several death-related pathways, robust ferroptosis was not achieved. To address this, we developed a biomimetic nanoagent, m(E@BN), composed of erastin-loaded boron nitride nanosheets coated with tumor cell membranes for targeted delivery. This system significantly enhanced BNCT-induced ferroptosis in vitro and in vivo, synergistically promoting lipid peroxidation and iron metabolism dysregulation without aggravating DNA injury. Furthermore, m(E@BN)-augmented BNCT stimulated potent antitumor immunity, evidenced by increased T cell infiltration and establishment of immune memory. Our study not only elucidates a mechanistic limitation of BNCT but also offers an effective nanomedicine-based strategy to amplify its efficacy through ferroptosis activation, providing a broadly applicable paradigm for improving radiotherapeutic outcomes.

## Introduction

Boron neutron capture therapy (BNCT) is an advanced radiotherapy strategy based on a binary targeting mechanism [1]. This technique involves injecting a ^10^B-containing drug into the patient's body, causing it to selectively accumulate within tumor cells. Then, thermal neutron beams are used to locally irradiate the tumor area. Neutrons undergo ^10^B(n,α)^7^Li capture reactions with ^10^B nuclei, generating α particles and ^7^Li recoil nuclei with high linear energy transfer (LET) properties [2]. These particles have an extremely short range in tissue (approximately 5 to 9 μm, equivalent to the diameter of a cell), enabling precise targeting of boron-enriched tumor cells while maximally sparing surrounding normal tissue [3,4]. However, there is still substantial heterogeneity in the clinical efficacy of BNCT. This is primarily due to uneven neutron flux distribution caused by differences in tissue density and composition, as [such as boronophenylalanine (BPA) and sodium borocaptate (BSH)] in the complex tumor microenvironment, which hinders the effectiveness of BNCT [3,5–7].

Current strategies to improve the efficacy of BNCT primarily focus on boron drug design and delivery innovations, such as developing novel boron-containing molecules and constructing new boron carriers with enhanced tumor selectively and tumor accumulation. Examples for boron-containing molecules include boron-containing nucleic acid analogs that can target the cell nucleus [8,9], amino acid derivatives (such as BPA and its glycosylated variants) that rely on metabolic competition [10], and porphyrin macrocyclic compounds with high boron loading capacity and natural tumor affinity [11,12]. Carriers such as liposomes [13], polymer micelles [14], and inorganic nanomaterials (such as functionalized boron carbon quantum dots [15] and boron nitride nanotubes [16,17]) not only improve the solubility and stability of boron drugs. Moreover, boron drugs effectively enhance their accumulation and retention in tumor tissues through surface functionalization (e.g., targeting ligands such as folic acid [18,19] and transferrin [20]) or biomimetic coating of tumor cell membranes [21], laying the foundation for more uniform and efficient boron distribution.

Despite the clinical prospects of BNCT, there are still differences in persistent response among different tumor types and patients, reflecting the inherent heterogeneity of boron pharmacokinetics and microregional therapeutic biology. Because BNCT is a high LET mode, where the ^10^B(n,α)^7^Li reaction produces dense ionized particles, resulting in complex DNA and macromolecular damage, tumor cells may experience different fates, including apoptosis, necrosis, autophagy, and mitotic disasters, depending on the tumor environment and stress adaptation status. Therefore, determining the main cell death program induced by BNCT is not only informative in mechanism but also critical in treatment: Firstly, different death modes eliminate drug-resistant subgroups differently, thereby forming response heterogeneity and recurrence risk; secondly, clear mechanisms are needed to design reasonable combinations to amplify the most effective pathways of death; and thirdly, the quality of cell death can determine immune consequences, as BNCT has been reported to induce immunogenic tumor cell death and reshape the tumor immune microenvironment, and combined strategies can further enhance systemic response.

In this study, we employed proteomic profiling to investigate the cell death mechanisms activated in response to BNCT. Results indicated that insufficient ferroptosis induction is a major contributing factor that may limit the therapeutic potential of BNCT. To evaluate this possibility, we engineered a tumor-targeted nanodelivery system, designated m(E@BN), which encapsulates the ferroptosis inducer erastin within a boron nitride nanocarrier, further functionalized with a homologous tumor cell membrane coating for enhanced targeting. Proteomic and functional analyses confirmed that the m(E@BN) system potently augments ferroptosis activation in tumor cells following BNCT, leading to significantly enhanced tumor cell eradication. In vivo experiments showed that m(E@BN) exhibited high tumor-selective accumulation in tumor-bearing mice (tumor versus normal tissue ratio = 30.69, tumor versus blood ratio = 15.17) due to its homologous targeting ability, significantly down-regulated GPX4 protein expression, activated CD4^+^/CD8^+^ T cell immunity, and induced long-term immune memory. In summary, this study not only delineates the cell death mechanisms in tumor cells following BNCT but also highlights the crucial role of ferroptosis in potentiating BNCT efficacy, thereby offering a novel therapeutic strategy and mechanistic insight for enhancing the effectiveness of BNCT.

## Materials and Methods

### Experimental design

The aim of this study is to verify the core hypothesis that “ferroptosis activation can enhance the efficacy of BNCT” and elucidate its potential mechanism. At the same time, we will develop a biomimetic nanoparticle [m(E@BN)] that integrates boron loading, ferroptosis induction, and homologous targeting functions. This formulation achieves ferroptosis activation by encapsulating the ferroptosis inducer erastin and homologous targeting through 4T1 tumor cell membrane encapsulation. We will systematically evaluate its tumor killing and immune activation ability from the dimensions of mechanism exploration, material construction, in vitro validation, and in vivo evaluation. Firstly, through multi-cell proteomics analysis and pathway enrichment, we will clarify the core mechanism of insufficient ferroptosis induction in BNCT monotherapy. Subsequently, the construction and characterization of m(E@BN) were completed, including the complete preparation process of h-BNNs synthesis, erastin loading, and tumor cell membrane encapsulation. The particle size, morphology, and chemical structure were characterized by dynamic light scattering (DLS), transmission electron microscopy (TEM), Fourier transform infrared spectroscopy (FTIR), and other methods, while the boron loading efficiency and erastin encapsulation efficiency were determined. Subsequently, in vitro experiments were conducted to evaluate the cell survival inhibition effect using Cell Counting Kit-8 (CCK-8) method and clone formation assay. DNA damage levels were detected by immunofluorescence of 53BP1, XRCC1, and OGG1, and changes in ferroptosis-related pathways were analyzed in combination with proteomic data. Finally, in the in vivo experiment, inductively coupled plasma mass spectrometry (ICP-MS) was used to detect the biological distribution of m(E@BN) in 4T1 tumor-bearing mice after intraperitoneal injection, confirming its targeted enrichment ability in tumor tissues. The antitumor effect was reevaluated in vivo, starting from the 9th day, and the tumor volume and mouse body weight were recorded every 2 d. The results show that the m(E@BN) + N group has the most significant tumor inhibitory effect, and hematoxylin and eosin (H&E) staining observed sparse arrangement and extensive necrosis of tumor cells in this group. Ki67 staining confirmed a decrease in tumor cell proliferation activity. In addition, we validated ferroptosis activation by detecting the down-regulation level of GPX4 protein through immunohistochemistry, and analyzed T cell infiltration in tumor tissues using flow cytometry and immunofluorescence. We also evaluated the establishment of immune memory through adoptive transfer experiments to comprehensively reveal the mechanism of action of this combination therapy.

### Materials

Cell culture medium (RPMI 1640, Dulbecco’s modified Eagle’s medium) and penicillin–streptomycin mixed solution (100×) were purchased from Meilun Biotechnology. Urea and boric acid were purchased from Aladdin. Anti-fluorescence quenching mounting solution [containing 4′,6-diamidino-2-phenylindole (DAPI)], cell membrane protein and cytoplasmic protein extraction kits, and bicinchoninic acid (BCA) protein concentration assay kits were purchased from Shanghai Biyuntian Biotechnology Co. Ltd. Rhodamine B, DiR (1,1'-dioctadecyl-3,3,3',3'-tetramethylindotricarbocyanine iodide), and polyvinyl alcohol (PVA) were purchased from MedChemExpress. Crystal violet was purchased from Yisheng Biotechnology Co. Ltd. Ethanol (99%), methanol, and dimethyl sulfoxide were purchased from Sinopharm Chemical Reagent Co. Ltd. Protein phosphatase inhibitors and CCK-8 detection kits were purchased from Beijing Solebo Technology Co. Ltd. Anti-mouse CD4 and anti-mouse CD8 antibodies, OGG1, XRCC1, and 53BP1 were purchased from Wuhan Saiwei Biotechnology Co. Ltd. (China). Unless otherwise stated, all antibodies were diluted 200× before use.

### Synthesis and characterization of m(E@BN)

PVA powder (240 mg) was dissolved in 6 ml of deionized water and stirred at 65 °C and 600 rpm until completely dissolved, preparing a 40 mg/ml PVA solution. This solution was added to the BN nanosheet dispersion, maintaining a BN:PVA mass ratio of 1:20, and sonicated for 2 h to achieve complete complexation. The complex was centrifuged at 12,000*g* for 10 min, and the precipitate was collected to obtain BN@PVA.

The BN@PVA complex was co-mixed with erastin powder for 24 h to fully load the erastin, resulting in E@BN nanoparticles. 4T1 mouse breast cancer cell membranes were extracted using a cell membrane protein extraction kit, mixed with E@BN nanoparticles, sonicated in a water bath for 3 to 5 min, and centrifuged at 10,000*g* for 15 min at 4 °C. The precipitate was collected to obtain 4T1 cell membrane-coated m(E@BN) nanoboron agents. The morphology of BNs was investigated using a transmission electron microscope (HT7800, Hitachi, Japan). The hydrodynamic size, polydispersity index (PDI), and zeta potential of the nanoparticles were recorded using a DLS instrument (Zetasizer, Malvern Instruments, UK) at 25 °C. FTIR was used to analyze the functional group composition and chemical structure within the wavelength range of 4,000 to 400 cm^−1^ at a resolution of 2 cm^−1^.

### Cellular uptake assay

4T1 cells in logarithmic growth phase were seeded at an appropriate density in confocal microplates. After the cells attached, RhoB-labeled BN nanoparticles were added and incubated in the dark for 2 h. The culture medium was discarded, and the cells were gently washed 3 times with ice-cold phosphate-buffered saline (PBS) to remove uninternalized nanoparticles. The cells were fixed with 4% paraformaldehyde for 15 min and rinsed with PBS, and nuclear staining with DAPI was performed. Finally, images were acquired using a laser scanning confocal microscope (CLSM).

### Inductively coupled plasma mass spectrometry

Boron content in cells, mouse tumors, muscle, and blood was measured using ICP-MS (PerkinElmer, NexION 300D, USA). Concentrated nitric acid (70%) was added and digested using a high-efficiency microwave digestion system before sample analysis.

### Animal experimentation

Female BALB/c mice (6 to 8 weeks old) were provided by Weitonglihua Laboratory Animal Technology Co. Ltd. (Beijing, China). All animal procedures were approved by the Laboratory Animal Care Group of the Institute of High Energy Physics, Chinese Academy of Sciences. All mice had free access to food.

4T1 cells (1 × 10^6^) suspended in PBS were inoculated subcutaneously into the right hind limb of mice to establish a 4T1 subcutaneous breast cancer model. Animal endpoints were evaluated in accordance with the “Guidelines for the Evaluation of Humane Endpoints in Laboratory Animals” (RB/T 173-2018). Mice were sacrificed when tumor size reached 2,500 mm^3^ or when they died due to severe weight loss.

### In vivo antitumor test

Tumor-bearing mice were randomly divided into 5 groups (*n* = 5): (a) PBS control group; (b) BN + N group; (c) m(E@BN) + N group. The corresponding preparations were injected through the tail vein, and neutron irradiation was performed 6 h later. Tumor volume and mouse body weight were measured every 2 d from the day of irradiation. After treatment, the mice were euthanized, and tumor tissues and spleens were obtained and digested with dissociation buffer at 37 °C for 2 h. Single-cell suspensions were prepared by filtration through a 70-μm filter. After washing with PBS, surface staining was performed using anti-CD4 and anti-CD8 antibodies, and the proportion of T cell subsets was detected by flow cytometry.

### Splenocyte adoptive transfer experiment

The spleens of treated mice were obtained, and spleen cell suspensions were prepared by grinding. After red blood cell lysis and resuspending in PBS, 1 × 10^5^ spleen cells were transferred into normal mice through tail vein injection. The recipient mice were designated as the PBS adoptive group and the m(E@BN) adoptive group based on spleen origin. Three days later, 2 × 10^5^ 4T1 cells were subcutaneously inoculated into the right hind limb of the recipient mice. Tumor growth was observed, and intratumoral T cell infiltration was analyzed.

### Tumor tissue immunofluorescence analysis

4T1 tumor-bearing mice were sacrificed after receiving the different treatments, and tumor tissue was excised. CD4 and CD8 immunofluorescence imaging was performed according to standard protocols. Representative images of sections imaged at the same magnification were used for analysis and analyzed in ImageJ. Frequency was defined as the percentage of positive cells in a given cell type.

### Statistical analysis

Experimental data are expressed as mean ± standard deviation (SD). Comparisons between 2 groups were performed using Student's *t* test, and multiple groups were analyzed using one-way analysis of variance (ANOVA). ns, not significant; *P < 0.05; **P < 0.01; ***P < 0.005; ****P < 0.001. GraphPad Prism 8 and Origin 2025 were used for data analysis and graphing, and flow cytometry data were processed using FlowJo software.

## Results

### Insufficient induction of ferroptosis by BNCT limits its therapeutic effect

**Fig. 1.**
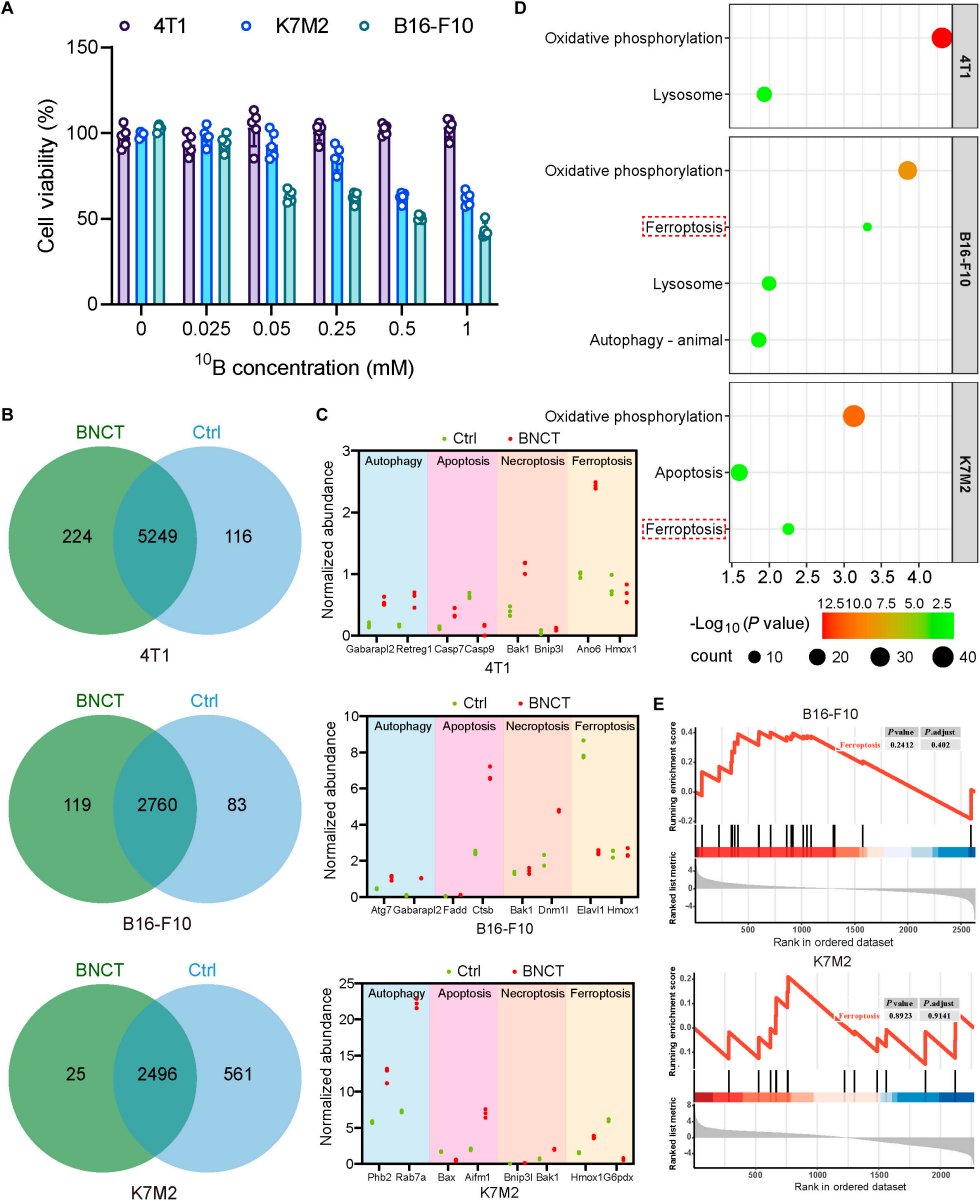
Insufficient induction of ferroptosis by BNCT limits its therapeutic effect. (A) Cell survival rates across experimental groups. (B) Venn diagram illustrating differentially expressed proteins. (C) Cell death-associated proteins in each experimental group. (D) Enriched cell death-related pathways in each experimental group. (E) GSEA analysis of ferroptosis pathways in each experimental group.

The goal of all radiotherapy, including BNCT, is to inhibit tumor cell proliferation and induce their death. Different cell death mechanisms lead to distinct final outcomes and contribute differently to treatment. Therefore, elucidating the mechanisms of cell death is crucial for radiotherapy. However, the specific mechanisms of tumor cell death following BNCT remain unclear. To address this, we collected cell samples from 3 tumor cell lines (breast cancer 4T1, melanoma B16F10, osteosarcoma K7M2) treated with BNCT. CCK-8 assay results showed that under different ^10^B concentrations, cell survival rates varied at 72 h post-irradiation. Specifically, B16F10 and K7M2 had survival rates close to 50% at 1 mM ^10^B, while 4T1 was less sensitive to BNCT, with a survival rate still near 100% (Fig. 1A). To investigate the underlying mechanisms, we further conducted liquid chromatography–tandem mass spectrometry (LC-MS/MS) proteomic analysis. The results indicated that most proteins identified in BNCT-treated groups were shared with control groups: 4T1 cells expressed 5,589 proteins (5,249 shared), B16-F10 cells expressed 2,962 proteins (2,760 shared), and K7M2 cells expressed 3,082 proteins (2,496 shared) (Fig. 1B). Further differential expression analysis of these shared proteins revealed changes in key proteins involved in multiple cell death pathways after BNCT, including autophagy, apoptosis, necroptosis, and ferroptosis (Fig. 1C and Fig. S1). Based on the Kyoto Encyclopedia of Genes and Genomes (KEGG) pathway enrichment analysis of differentially expressed proteins, it was found that the oxidative phosphorylation pathway was the most enriched and statistically significant in all 3 cell lines (Fig. 1D), suggesting that mitochondrial dysfunction might be the core event following BNCT treatment. Considering cell death mechanisms, mitochondrial dysfunction is typically associated with various death pathways, including apoptosis, necroptosis, and ferroptosis. Our KEGG pathway enrichment analysis of the 3 cell lines revealed that no ferroptosis pathway was detected in 4T1 cells, while ferroptosis pathways were enriched in the B16F10 and K7M2 cell lines (Fig. 1D). This finding aligns with the cell survival rates of the 3 cells following BNCT, suggesting that ferroptosis may be related to the survival rates of tumor cells after BNCT. Through Gene Set Enrichment Analysis (GSEA) on the ferroptosis pathways of B16F10 and K7M2 cells, it was found that ferroptosis in B16F10 cells [normalized enrichment score (NES) = 1.17, *P* = 0.24] and K7M2 cells (NES = 0.67, *P* = 0.89) was below the threshold (NES serves as a key metric for evaluating the enrichment level of a specific gene set within a list of genes ranked by phenotypic association [22]; a |NES| > 1.5 indicates significant enrichment of the gene set in the phenotype, suggesting that the pathway as a whole is in an activated state [23]), indicating that ferroptosis was not markedly activated (Fig. 1E). This suggests that BNCT is not strongly effective in inducing ferroptosis.

Traditional views hold that radiotherapy primarily induces cell apoptosis by directly damaging DNA (e.g., causing double-strand breaks) [24]. However, recent studies have shown that radiotherapy can synergize with multiple cell death mechanisms, and its ultimate efficacy is closely related to the induced cell death patterns and their immunogenicity. Ferroptosis—a form of iron-dependent, lipid peroxidation-driven programmed cell death—has garnered attention due to its strong synergy with radiotherapy [25]. This process is initiated by iron ions catalyzing the peroxidation of polyunsaturated fatty acids, ultimately leading to cell membrane rupture. The core regulatory mechanisms involve antioxidant pathways such as GPX4 and System Xc^−^ [26,27]. Ferroptosis can not only eliminate apoptosis-resistant tumor stem cell subpopulations but also release danger signals like HMGB1 and ATP (adenosine triphosphate), exhibiting immunogenic cell death characteristics, which promote dendritic cell maturation and T cell activation [28]. Therefore, combining drugs that induce ferroptosis (such as erastin and RSL3) with radiotherapy is expected to enhance local killing effects and elicit systemic antitumor immunity [29,30]. Based on this, we propose a scientific hypothesis: Activating ferroptosis in tumor cells treated with BNCT could further improve the efficacy of BNCT radiotherapy and immune activation.

### Synthesis and characterization of ferroptosis inducer-loaded nanoparticles m(E@BN)

**Fig. 2.**
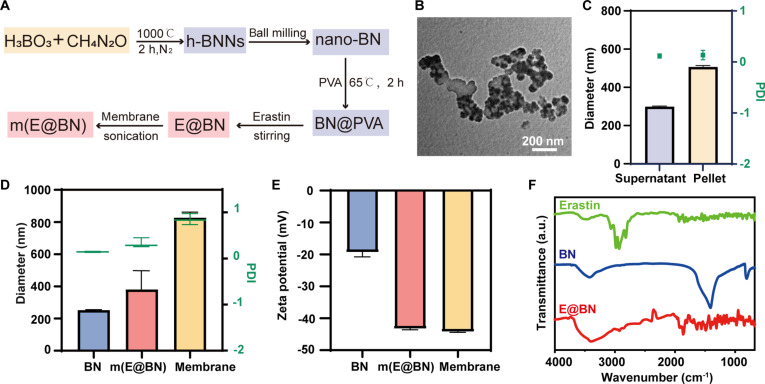
Characterization of m(E@BN) nanoboron agents. (A) Schematic diagram of the synthesis route of m(E@BN). (B) TEM image of BN. Scale bar, 200 nm. (C) Hydration particle size and PDI of the supernatant and precipitate after ball milling and centrifugation. (D and E) Hydration particle size, PDI, and zeta potential of BN, m(E@BN), and cell membrane. (F) FTIR of erastin, BN, and E@BN.

To validate this hypothesis, we first designed a ^10^B drug delivery system loaded with the ferroptosis inducer erastin. As a classic inducer, erastin inhibits the System Xc^−^ (cystine/glutamate antiporter dimer) channel to deplete glutathione, thereby disrupting antioxidant defenses and efficiently inducing ferroptosis [31,32]. The synthesis process of m(E@BN) is shown in Fig. 2A. Hexagonal boron nitride nanosheets (h-BNNs) were first prepared via a solvent-mediated lattice control strategy. The h-BNNs were obtained by recrystallization in a low-boiling-point methanol–water solution, followed by thermal decomposition of a eutectic mixture of urea and boric acid at 1,000 °C. Then, they were subjected to ball milling treatment to transform into nano-BN. TEM images (Fig. 2B) show that the prepared BN exhibits a hexagonal sheet structure with a size of approximately 50 nm. After mixing the nano-BN with the PVA solution, it was centrifuged at 1,800*g* for 10 min. The supernatant and precipitate were collected respectively for DLS detection. As shown in Fig. 2C, the hydrated particle size of the supernatant is smaller than pellet. Therefore, we selected the supernatant and continued to centrifuge 12,000*g* for 10 min to collect the pellet as BN@PVA.

The high surface area and mesoporous structure of BN facilitate drug loading. 4T1 tumor cell membranes were further coated on the E@BN surface by water bath sonication, yielding m(E@BN). DLS analysis showed that the average hydrodynamic diameter of m(E@BN) was 383.57 ± 91.08 nm, larger than the 251.8 ± 3.13 nm of BN and smaller than the 826.8 ± 28.59 nm of the membrane (Fig. 2D). Its zeta potential was −43.4 ± 0.2 mV, close to the −43.95 ± 0.15mV of the cell membrane (Fig. 2E), indicating successful cell membrane encapsulation. Erastin was stirred with BN@PVA for 24 h and then centrifuged to collect the pellet as E@BN. FTIR revealed that the absorption peak of E@BN at 1,382 cm^−1^ was attributed to the in-plane stretching vibration of h-BN, while the absorption peak at 2,927 cm^−1^ indicated successful erastin loading (Fig. 2F). Quantitative analysis revealed an erastin loading efficiency of ~67% for m(E@BN), with sustained release kinetics in PBS (pH 7.4) showing a cumulative release of 55.29 ± 3.66% at 24 h (Fig. S2). In conclusion, we have successfully synthesized BN nanoparticles coated on tumor cell membranes and loaded with erastin.

### Evaluation of the in vitro BNCT effect of m(E@BN)

To verify the homologous targeting ability of nanoparticles m(E@BN) coated on the membrane of 4T1 cells, we compared the uptake efficiency of DiR-labeled m(E@BN) in homologous cells (4T1) and nonhomologous cells (143B), as well as the uptake of m(E@BN) coated on nonhomologous K7M2 membrane in 4T1 cells. The results showed that the uptake efficiency of homologous pairing was remarkably higher than that of the 2 nonhomologous combinations (Fig. S3), confirming that homologous membrane encapsulation can enhance the specific uptake ability of target cells.

**Fig. 3.**
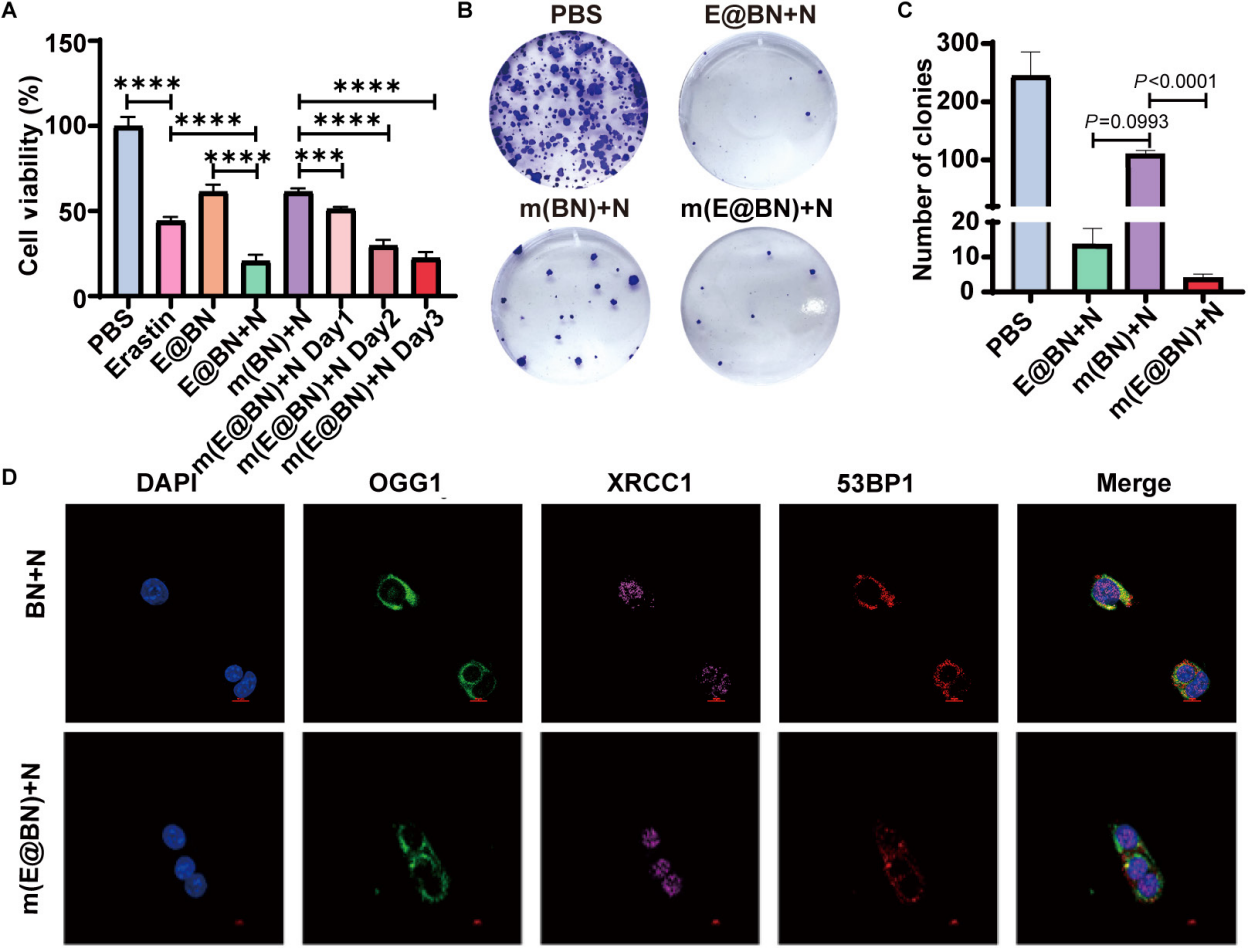
Evaluation of the in vitro killing effect of m(E@BN) BNCT. (A) 4T1 cell viability in each group. (B and C) Graphs of clonogenic assay results and number of colonies in each group. (D) Immunofluorescence staining of DNA damage markers OGG1 (green), XRCC1 (purple), and 53BP1 (red) after neutron irradiation. Data are presented as mean ± SD. Statistical significance was obtained by 2-sided Student's *t* test and one-way ANOVA. *P < 0.05, **P < 0.01, ***P < 0.005, ****P < 0.001.

Subsequently, we evaluated the effects of PBS, E@BN, m(BN), and m(E@BN) on 4T1 cell survival under neutron irradiation. CCK-8 assay results showed that cell viability decreased in the erastin, E@BN, m(BN), and m(E@BN) treatment groups compared to the PBS group. Notably, the cell killing ability of the E@BN + N group was significantly higher than that of the erastin group and the E@BN group (Fig. 3A). Clonogenicity assays further validated the BNCT efficiency of m(E@BN) under neutron irradiation (Fig. 3B and C). The number of cell colonies in the E@BN + N and m(E@BN) + N groups was significantly lower than that in the PBS and m(BN) + N groups, indicating that erastin loading further enhanced cell killing.

High-LET particles during BNCT can induce multiple DNA lesions, including double-strand breaks (DSBs), single-strand breaks (SSBs), and base lesions. To evaluate the effect of m(E@BN) + N treatment on the DNA damage response, we performed immunofluorescence staining for OGG1 (base lesions), XRCC1 (SSBs), and 53BP1 (DSBs) in 4T1 cells 24 h after neutron irradiation (Fig. 3D). There was no difference in the fluorescence signals of OGG1, XRCC1, and 53BP1 in BN + N and m(E@BN) + N groups (Fig. S4), indicating that the addition of erastin did not cause additional load on the base lesions or SSB or DSB repair pathways examined. It suggests that erastin mainly enhances the antitumor effect of radiotherapy through the ferroptosis pathway.

### m(E@BN) drives robust ferroptosis after BNCT

**Fig. 4.**
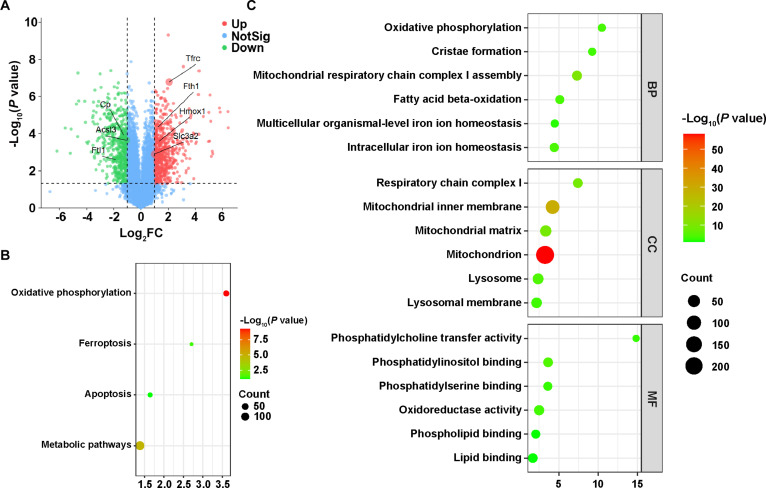
m(E@BN) drives robust ferroptosis after BNCT. (A) Volcano plot displaying differentially expressed proteins. (B) KEGG analysis of significantly differentially expressed proteins between m(E@BN)-based BNCT and the control group, and (C) GO enrichment analysis for biological process (BP), cellular component (CC), and molecular function (MF).

We used the 4T1 cell line, where ferroptosis activation by BNCT is least pronounced, to validate the role of ferroptosis inducer-loaded nanoparticles [m(E@BN)] in inducing ferroptosis after BNCT treatment. The results showed that the ferroptosis pathway and oxidative phosphorylation pathway were significantly activated (Fig. 4A and B and Fig. S5). Further analysis revealed that the ferroptosis pathway enriched key upstream molecules including System Xc^−^, ACSL4, CP, TFR1, and HO-1, suggesting that m(E@BN) + N treatment effectively initiates ferroptosis. These molecules play central roles in iron metabolism and lipid peroxidation: The Xc^−^ transporter regulates cysteine uptake, influencing glutathione levels and thereby determining cellular sensitivity to oxidative damage and ferroptosis; ACSL4 promotes polyunsaturated fatty acid esterification, supplying substrates for ferroptosis-associated lipid peroxidation reactions; while CP, TFR1, and HO-1 synergistically regulate cellular iron influx, oxidation, and heme degradation, promoting intracellular free iron accumulation, enhancing the Fenton reaction, and ultimately exacerbating lipid peroxidation and cell death (Fig. S6). Additionally, intracellular iron ion homeostasis (*P* = 7.48E−05, Fold = 4.42) and multicellular organismal-level iron ion homeostasis (*P* = 0.01, Fold = 4.49) pathways were activated considerably, suggesting that BNCT + E treatment promotes iron mobilization, providing the necessary “iron source” for ferroptosis (Fig. 4C). Relevant proteins involved in iron regulation included ferritin (FTH1/FTL), NCOA4 (ferritin autophagy), and SLC40A1/SLC39A14 (iron export/import).

Significant activation of oxidative phosphorylation (*P* = 1.75E−04, Fold = 10.47), fatty acid β-oxidation (*P* = 1.19E−04, Fold = 5.13), respiratory chain complex I (*P* = 2.01E−09, Fold = 7.42), mitochondrion (*P* = 1.00E−58, Fold = 3.26), and mitochondrial inner membrane (*P* = 4.84E−30, Fold = 4.21) was observed, which aligns with the characteristic features of lipid peroxidation in ferroptosis. Furthermore, cristae formation (*P* = 3.38E−04, Fold = 9.24) was activated, indicating that mitochondrial dysfunction exacerbates lipid peroxidation (Fig. 4C). Overall, the combined treatment of BNCT with erastin efficiently coupled iron flow, lipid metabolism, and oxidative stress in specific cellular compartments, ultimately driving ferroptosis.

In addition, to functionally validate the core role of ferroptosis in enhancing the efficacy of BNCT, we used ferroptosis inhibitors [Ferrostatin-1 (Fer-1)]. The result indicates that pretreatment with Fer-1 (6 μM) for 4 h or combined treatment (co-treatment) can effectively reverse the cytotoxicity induced by m(E@BN) + N, confirming that ferroptosis is the main mode of death in combination therapy. On the contrary, Fer-1 failed to protect cells from m(BN) + N-induced cell death, indicating that BNCT alone does not primarily kill through ferroptosis (Fig. S7). This stark contrast functionally validates our proteomic observations: BNCT has insufficient activation of ferroptosis, while the introduction of erastin shifts the cell death mechanism toward and becomes sensitive to this pathway. Therefore, activating ferroptosis can causally enhance the efficacy of BNCT.

### m(E@BN) inhibits tumor growth in 4T1 breast cancer mice

**Fig. 5.**
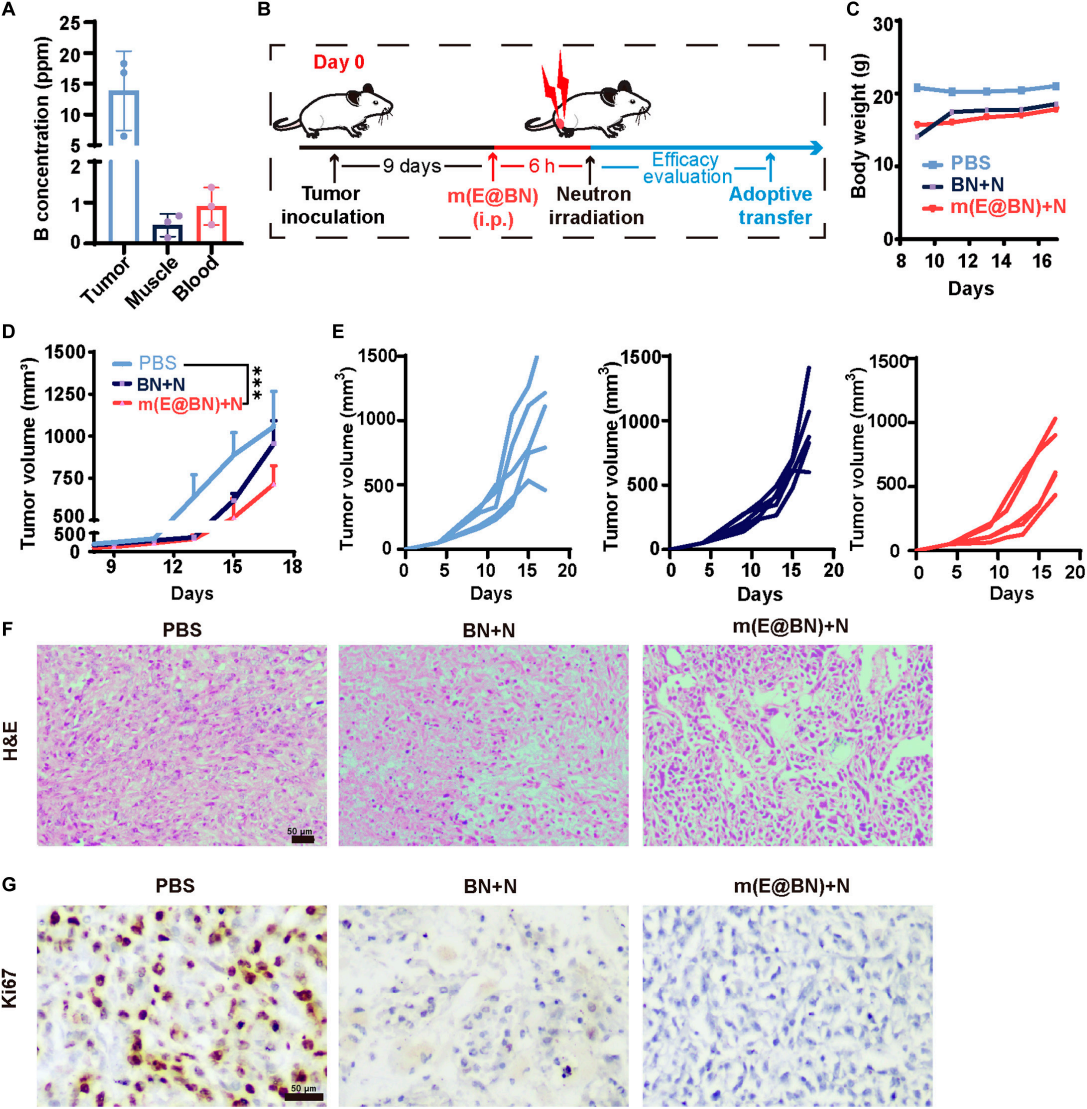
m(E@BN) inhibits tumor growth in 4T1 breast cancer mice. (A) Boron levels in mouse tumors, muscle, and blood 6 h after injection with PBS, BN, and m(E@BN). (B) Schematic diagram of the animal experiment process. (C) Body weight changes of mice in the PBS, BN + N, and m(E@BN) + N groups after treatment. (D and E) Tumor growth curves of mice in different groups. (F and G) H&E and Ki67 staining of tumor tissues. Data are presented as mean ± SD. Scale bar, 50 μm. Statistical significance was obtained by 2-sided Student's *t* test and one-way ANOVA. *P < 0.05, **P < 0.01, ***P < 0.005, ****P < 0.001.

Prior to evaluating the therapeutic efficacy of m(E@BN) nanoparticles (NPs) in vivo, the biodistribution performance of NPs was investigated using a 4T1 tumor-bearing mouse model. We detected the B content by ICP-MS measurement to reflect the accumulation of m(E@BN) post-intraperitoneal injection. The accumulation of m(E@BN) in tumor tissues reached a maximum at 6 to 8 h post-injection at 13.86 ± 6.453 ppm (parts per million), significantly higher than levels in muscle (0.45 ± 0.23 ppm) and blood (0.91 ± 0.37 ppm) (Fig. 5A). Based on in vitro results, we evaluated the in vivo antitumor effect of m(E@BN) in a 4T1 tumor model (Fig. 5B). Drug injections were administered starting on day 9. Tumor volume and body weight were recorded every 2 d. Body weight curves showed no obvious changes among groups (Fig. 5C), indicating that m(E@BN) had no apparent systemic toxicity. Tumor growth curves revealed that the m(E@BN) + N group had the most significant tumor inhibition effect. Tumor growth inhibition (TGI) was 38.8% for the BN + N group and 54.8% for the m(E@BN) + N group (versus PBS) (Fig. 5D and E). The tumor tissues of the 3 groups were stained with H&E to observe their pathological changes (Fig. 5F). It was found that compared with PBS and BN + N groups, the tumor cells in the m(E@BN) + N group were sparsely arranged and there was extensive tumor cell necrosis. The proliferation level of tumor cells in tumor tissue was observed by Ki67 staining (Fig. 5G), and it was found that the expression of Ki67-positive cells (brown nuclei) in the m(E@BN) + N group was reduced compared with the PBS group. This showed that m(E@BN) reduced the proliferative activity of tumor cells after neutron irradiation, indicating that this treatment effectively inhibited tumor cell proliferation.

### m(E@BN) + N treatment activates antitumor immune responses

**Fig. 6.**
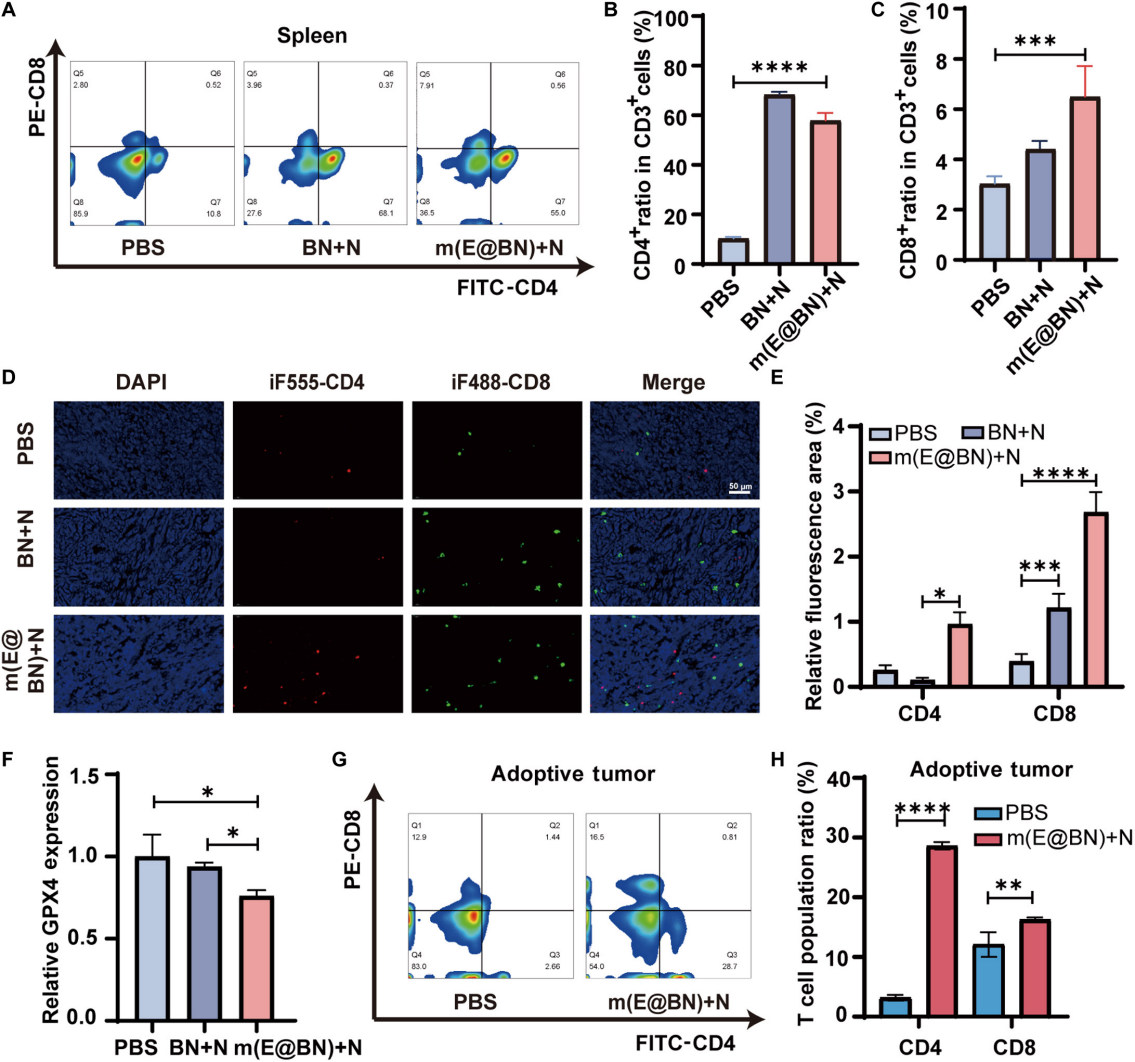
Antitumor immune effects in mice after m(E@BN) + N treatment. (A) Flow cytometry results showing the ratios of CD4^+^ and CD8^+^ T cells in the spleens of mice in the PBS, BN, and m(E@BN) groups. (B and C) Flow cytometric analysis of CD4^+^ and CD8^+^ T cells in the spleens of the 3 groups of mice. (D and E) Fluorescent staining of CD4^+^ and CD8^+^ T cells in tumor tissues. Scale bar, 50 μm. (F) Relative expression of GPX4 in the 3 groups of tumor tissues normalized by glyceraldehyde-3-phosphate dehydrogenase (GAPDH). (G and H) Flow cytometric results and statistics of CD4^+^ and CD8^+^ T cells in tumors of mice in the PBS and m(E@BN) + N adoptive transfer groups. Data are presented as mean ± SD. Statistical significance was obtained by 2-sided Student's *t* test and one-way ANOVA. *P < 0.05, **P < 0.01, ***P < 0.005, ****P < 0.001.

Some studies have shown that when tumor cells undergo ferroptosis, they can affect the tumor immune microenvironment by regulating immune cells, thereby activating the antitumor immune response [33,34]. To investigate whether m(E@BN)-mediated BNCT can stimulate antitumor immunity, we examined the proportion of T cells in the spleens of treated mice. Flow cytometry results (Fig. 6A to C) showed that the proportions of CD4^+^ and CD8^+^ T cells in the spleens of the m(E@BN) + N group were significantly higher than those in the PBS group. Immunofluorescence staining results showed that compared with the PBS group, the infiltration of CD4^+^ and CD8^+^ T cells in the tumor tissue of the m(E@BN) + N group was increased prominently (Fig. 6D and E), indicating that this treatment can effectively activate the antitumor T cell immune response. Immunohistochemistry showed that the GPX4-positive signal in mouse tumor tissue was tan and was mainly localized to the cytoplasm. Compared with the PBS group and the BN + N group, the positive area in the m(E@BN) + N group reduced, suggesting that GPX4 expression was down-regulated (Fig. S8). Western blot further confirmed that the GPX4 protein level in the BN + N group was 0.94-fold (about 6%) that in the PBS group, while the m(E@BN) + N group dropped to 0.76-fold (about 24%), suggesting that m(E@BN) triggers tumor cell ferroptosis by down-regulating GPX4, thereby activating the antitumor immune response (Fig. 6F). To further evaluate the immune memory effect, we performed a spleen cell adoptive transfer experiment. Splenocytes from mice in the PBS and m(E@BN) + N groups were adopted into normal mice, and 4T1 cells were inoculated 3 d later. Flow cytometry revealed that, compared to the PBS-transferred group, the m(E@BN)-transferred group exhibited significantly increased CD4^+^ and CD8^+^ T cell infiltration in tumor tissues as well as elevated frequency of central memory T cells (Tcm, CD44^+^CD62L^+^) in the spleen (Fig. 6G and H and Fig. S9), indicating that this treatment can induce long-term antitumor immune memory.

## Discussion

Despite decades of development, our understanding of how tumor cells die after BNCT remains limited. This contrasts with conventional radiotherapy, where DNA damage responses and apoptosis have been extensively characterized. BNCT generates high-LET α particles and ^7^Li recoil nuclei with cell-scale path lengths. As a result, the injury is not confined to nuclear DNA but can also affect cellular membranes and organelles. Therefore, it is important to determine which regulated cell death pathways are actually engaged by BNCT. Such knowledge is directly relevant to explaining response heterogeneity and treatment resistance, and ultimately to improving therapeutic efficacy.

As an iron-dependent, lipid peroxidation-driven regulatory cell death mechanism, ferroptosis relies on the accumulation of polyunsaturated fatty acid phospholipid (PUFA PL) peroxidation damage, while GPX4 antioxidant defense and FSP1 coenzyme Q10 (CoQ) axis constitute the main protective mechanisms of cells [35–38]. Ionizing radiation can induce ferroptosis through 3 pathways: reactive oxygen species (ROS) generation, ACSL4 up-regulation, and glutathione (GSH) depletion, and has a positive interaction with antitumor immunity. However, existing research has mostly focused on traditional photon radiotherapy [39–41], and there is still a lack of in-depth exploration of the regulatory mechanisms and therapeutic potential of ferroptosis in high LET radiotherapy such as BNCT. In our proteomic analyses across multiple tumor cell lines, mitochondrial stress emerged as a common response to BNCT and was accompanied by oxidative and metabolic stress conditions that can favor lipid peroxidation. However, accordingly, ferroptosis was not strongly activated. We therefore propose that insufficient ferroptosis engagement may represent an important factor that limits the therapeutic potential of BNCT and may attenuate overall efficacy.

Based on this rationale, we engineered a tumor-targeted nanodelivery system, m(E@BN), which encapsulates the ferroptosis inducer erastin within a boron nitride nanocarrier and is further functionalized with a homologous tumor cell membrane coating to enhance targeting. Consistent with this design, we observed increased ferroptosis, improved tumor control, and higher intratumoral infiltration of CD4^+^ and CD8^+^ T cells, together with memory-associated effects. These findings highlight the potential of modulating cell death pathways to improve radiotherapeutic outcomes and offer a translatable combinatorial paradigm for cancer treatment.

While our findings provide deeper mechanistic insight, several challenges remain that should be addressed in future studies. BNCT requires specialized infrastructure and qualified neutron sources, and neutron beam characteristics can differ across platforms and centers. Dosimetry workflows also vary, and boron pharmacokinetics may change across experimental and clinical settings. These factors can affect reproducibility and regulatory feasibility. Future work should therefore evaluate robustness across representative neutron source conditions and establish a translational framework covering manufacturing, stability, and safety. Finally, although tumor cell membrane coating enhances homologous targeting, it complicates scale-up and quality control. Key issues include membrane source consistency, batch reproducibility, and storage stability. Future designs should strengthen standardization criteria or adopt more scalable targeting strategies while preserving the core concept of boron–ferroptosis co-optimization.

## Data Availability

Data will be made available on request.
