# Supplementary material for: Boosting Boron Neutron Capture Therapy through Ferroptosis Activation with a Biomimetic Nanoenhancer
Source: Biomater Res. 2026 Apr 3;30:0343. doi: 10.34133/bmr.0343 (PMC13047272; doi:10.34133/bmr.0343)
Supplement: Supplementary 1 — Figs. S1 to S9 [file bmr.0343.f1.docx]

**SUPPLEMENTARY MATERIALS**

**Supplementary Methods**

**BN Nanosizing**

3 mg of hexagonal boron nitride (h-BN) powder was added to 400 μL of deionized water (DI) and 0.5 mL of large zirconia grinding beads (0.8-1.0 mm diameter) and ball-milled at 30 Hz for 15-20 minutes. The resulting suspension was transferred to a centrifuge tube and repeatedly washed with DI water. The combined samples were centrifuged at 12,000 g for 10 minutes at 25°C, and the supernatant discarded. 400 μL of DI water and 0.5 mL of small zirconia grinding beads (0.22 mm diameter) were added to the precipitate and ball-milled again for 30 minutes. The resulting suspension was collected and transferred to a 15 mL centrifuge tube, adjusted to a volume of 1 mg/mL for the BN nanosheet concentration. The beads were removed by filtration using a 70 μm cell strainer to obtain a uniform BN nanosheet suspension. The suspension was sterilized by autoclaving and stored in an UHV suitcase vacuum transfer device (CASAcme Technology, Huairou, Beijing) at 4°C until further use.

**Cell Viability Assay**

4T1 cells were seeded at a density of 5,000 cells per well in a 96-well plate. After adherence, cells were treated with PBS, E@BN, m(BN), and m(E@BN), followed by neutron irradiation. After continued culture for a specified period, CCK-8 solution was added to each well. After incubation for an appropriate period, absorbance at 450 nm was measured using a microplate reader to calculate relative cell viability.

**Colony formation assay**

4T1 cells were seeded at 1000 cells per well in 6-well plates and divided into PBS, BN+N, E@BN+N, and m(E@BN)+N groups, with triplicate wells per group. After 24 hours of treatment, cells were neutron-irradiated and cultured for 10 days after replacement with fresh medium. The medium was discarded, and cells were washed with PBS, fixed with 4% paraformaldehyde for 30 minutes, stained with 0.5% crystal violet for 5 minutes, and washed with PBS to clear background. Cell colonies were then photographed and counted using ImageJ software.

**Immunofluorescence Staining**

After different treatments, 4T1 cells were washed three times with P, fixed with 4% paraformaldehyde for 30 minutes, permeabilized with 0.1% Triton X-100 for 10 minutes, and nonspecific binding sites were blocked with 3% BSA. The corresponding primary antibodies (OGG1, XRCC1, 53BP1, CD4, and CD8) were then added and incubated at room temperature for 1-2 hours. After rinsing with PBS, fluorescent secondary antibodies were added and incubated in the dark for 30 minutes. Nuclei were stained with DAPI for 5 minutes. The slides were mounted and observed and photographed under a confocal microscope.

**Supplementary Figures**


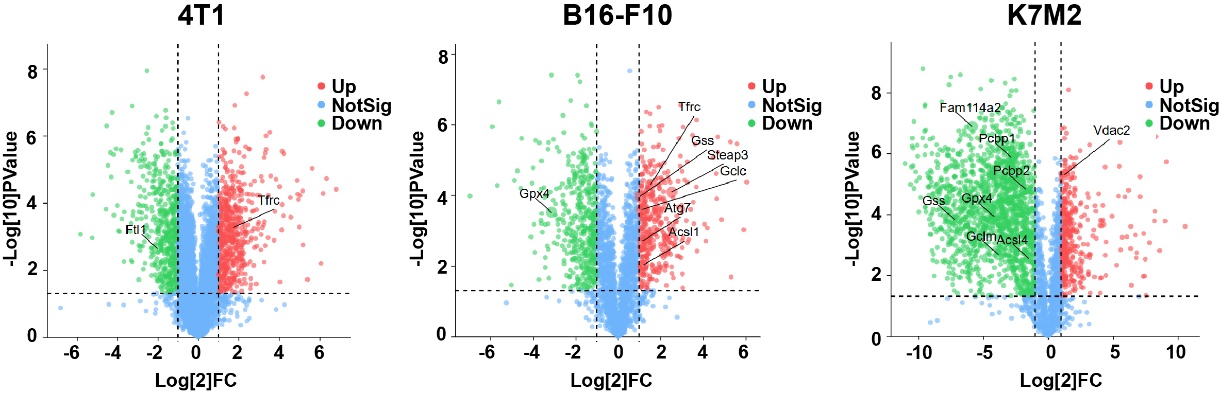


**Figure S1.** Volcano map shows the differential expression of shared proteins in three cell lines K7M2, B16-F10, and 4T1 after BNCT treatment (control group vs BNCT group).


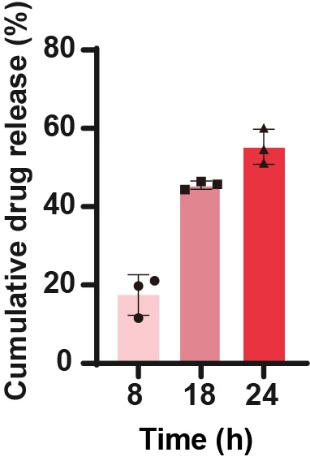


**Figure S2**. In vitro drug-release profile of m(E@BN) in PBS (pH 7.4) at 37 °C. Data are presented as mean ± SD (n = 3).


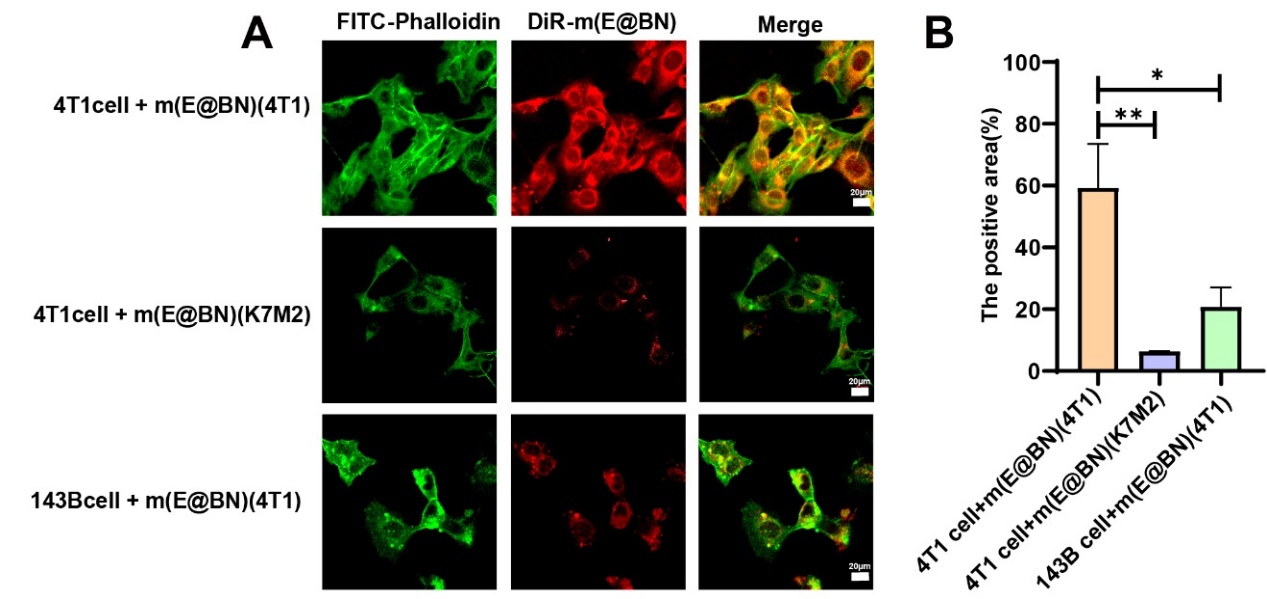


**Figure S3.** (A) Representative confocal microscopy images showing 4T1 cell membrane-coated m(E@BN) co-incubated with homologous 4T1 cells or non-homologous 143B cells, and K7M2 cell membrane-coated m(E@BN) co-incubated with 4T1 cells. Cytoskeleton was stained with FITC-phalloidin (green), and m(E@BN) was labeled with DIR dye (red). Scale bar: 20 μm. (B) Quantitative analysis of the average DIR fluorescence area in the fields of view from (A). Data are presented as mean ± standard deviation (SD); one-way ANOVA. * p < 0.05. ** p < 0.01.


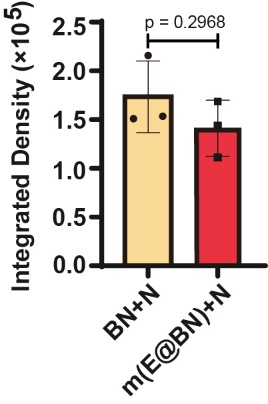


**Figure S4.** Integrated density of DNA damage markers in 4T1 cells treated with BN+N and m(E@BN)+N. The integrated densities of OGG1, XRCC1 and 53BP1 channels were summed by ImageJ. The data is mean ± SD; ns: p>0.05 by unpaired t-test, indicating no significant difference.


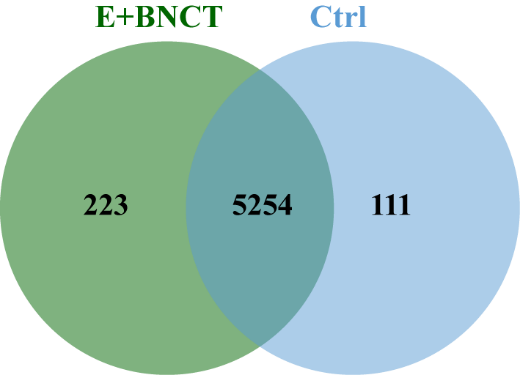


**Figure S5.** Venn diagram shows the distribution of differentially expressed proteins of 4T1 (BNCT+E group vs control group).


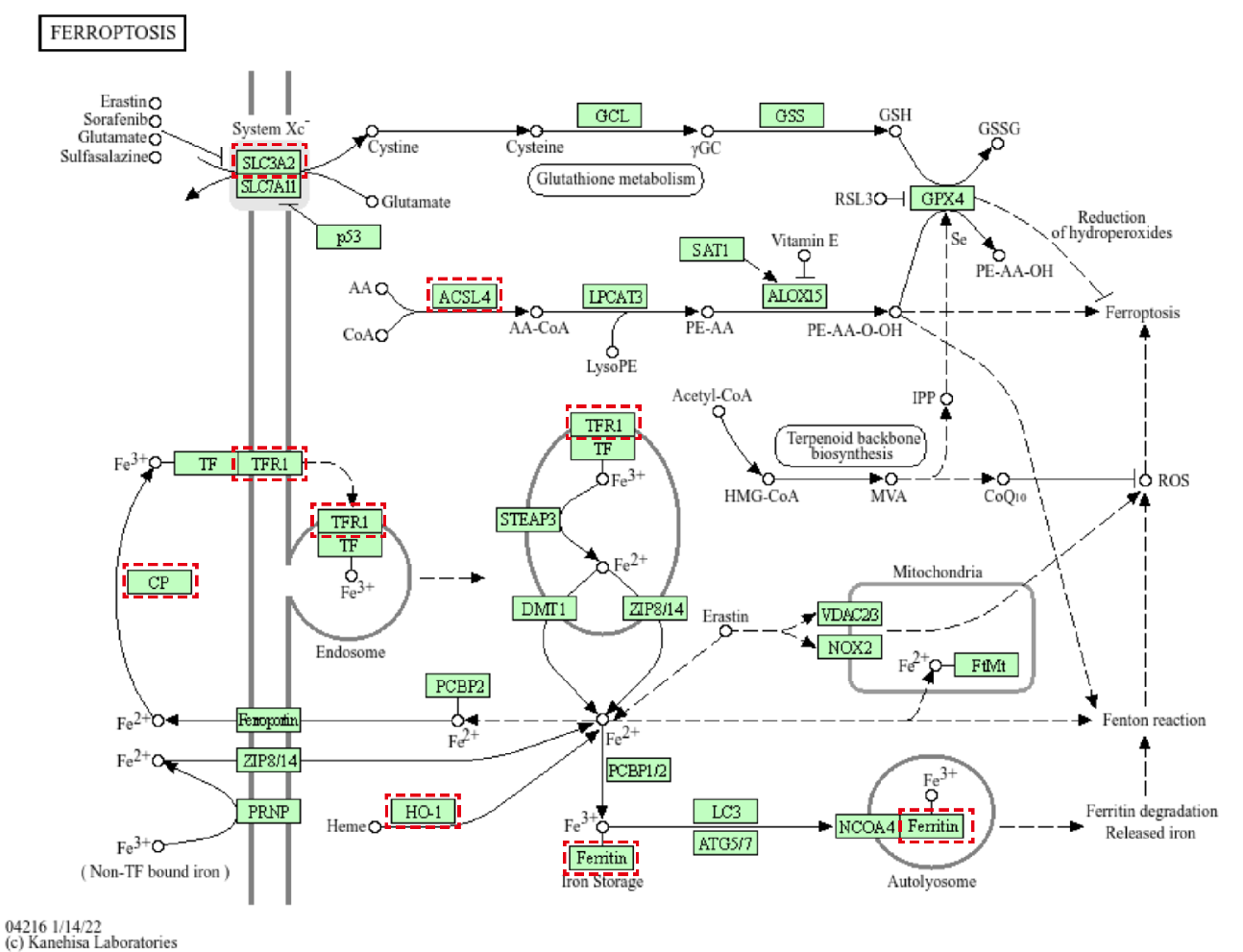


**Figure S6.** Activation of ferroptosis pathway protein after 4T1 cells after m(E@BN)+N treatment.


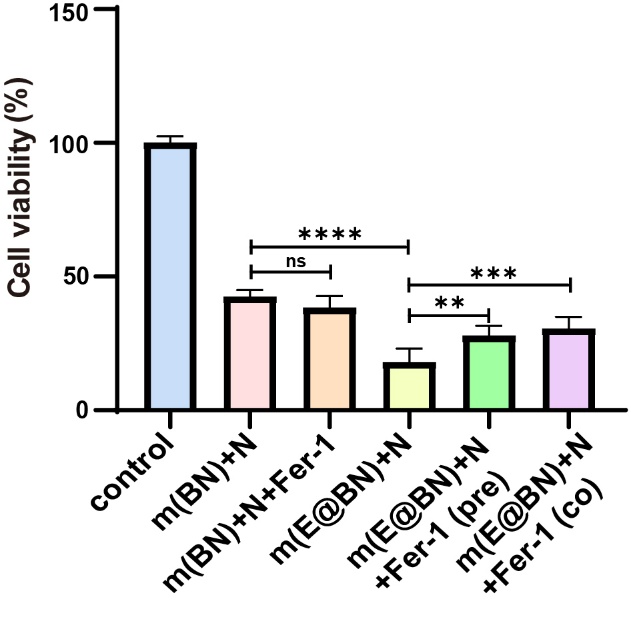


**Figure S7.** Fer-1 rescues cells from m(E@BN)+N-induced cytotoxicity. 4T1 cells were treated as follows: control (no treatment); m(BN)+N (BNCT alone); m(BN)+N+Fer-1 (BNCT with Fer-1 co-treatment); m(E@BN)+N (BNCT with ferroptosis induction); m(E@BN)+N+Fer-1(pre) (Fer-1 pretreatment for 4 h prior to m(E@BN)+N); or m(E@BN)+N+Fer-1(co) (Fer-1 co-treatment with m(E@BN)+N). Fer-1 concentration was 6 μM. Cell viability was measured by CCK-8 at 24 h post-neutron irradiation. Data are mean ± SD (n = 6); one-way ANOVA. **p < 0.01, ***p < 0.005, ****p < 0.0001, ns = not significant.


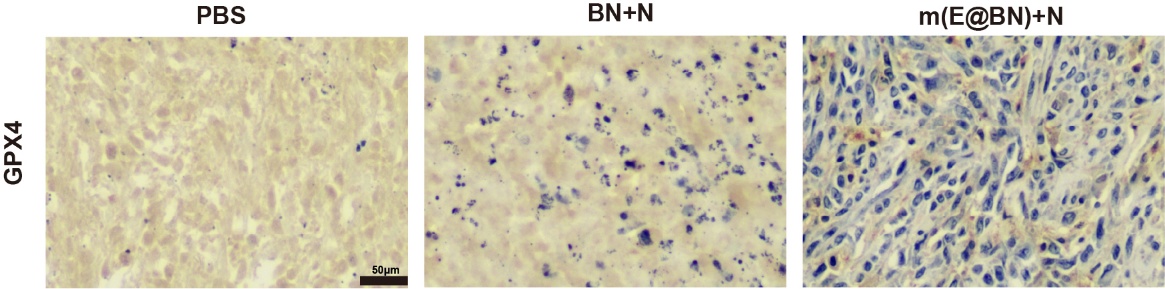


**Figure S8.** Representative immunohistochemical staining of GPX4 in tumor sections of three groups of mice. Brown signals indicate GPX4-positive cytoplasmic expression. Scale bar: 50 μm.


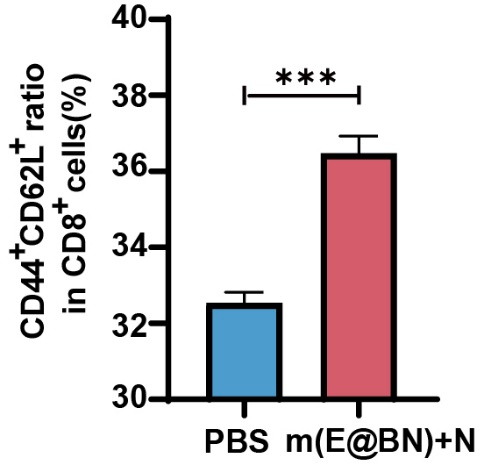


**Figure S9.** Recipient mice were intravenously injected with splenocytes from either PBS-treated control donors or m(E@BN)+N cured donors. Three days later, all recipient mice were challenged with 4T1 tumor cells. At the experimental endpoint (30 days after tumor inoculation), the proportion of central memory T cells(CD8⁺CD44⁺CD62L⁺ ) in the spleen was analyzed by flow cytometry. Data are presented as mean ± standard deviation (n = 5 mice per group). Statistical significance was determined using an unpaired two-tailed Student’s t-test (***p < 0.001).
